# Food Catches the Eye but Not for Everyone: A BMI–Contingent Attentional Bias in Rapid Detection of Nutriments

**DOI:** 10.1371/journal.pone.0019215

**Published:** 2011-05-16

**Authors:** Lauri Nummenmaa, Jari K. Hietanen, Manuel G. Calvo, Jukka Hyönä

**Affiliations:** 1 Brain Research Unit, Low Temperature Laboratory, Aalto University School of Science, Espoo, Finland; 2 Department of Biomedical Engineering and Computational Science, Aalto University School of Science, Espoo, Finland; 3 Turku PET Centre, University of Turku, Turku, Finland; 4 Department of Psychology, University of Tampere, Tampere, Finland; 5 Department of Cognitive Psychology, Universidad de La Laguna, La Laguna, Spain; 6 Department of Psychology, University of Turku, Turku, Finland; University of Regensburg, Germany

## Abstract

An organism's survival depends crucially on its ability to detect and acquire nutriment. Attention circuits interact with cognitive and motivational systems to facilitate detection of salient sensory events in the environment. Here we show that the human attentional system is tuned to detect food targets among nonfood items. In two visual search experiments participants searched for discrepant food targets embedded in an array of nonfood distracters or vice versa. Detection times were faster when targets were food rather than nonfood items, and the detection advantage for food items showed a significant negative correlation with Body Mass Index (BMI). Also, eye tracking during searching within arrays of visually homogenous food and nonfood targets demonstrated that the BMI-contingent attentional bias was due to rapid capturing of the eyes by food items in individuals with low BMI. However, BMI was not associated with decision times after the discrepant food item was fixated. The results suggest that visual attention is biased towards foods, and that individual differences in energy consumption - as indexed by BMI - are associated with differential attentional effects related to foods. We speculate that such differences may constitute an important risk factor for gaining weight.

## Introduction

Attention ensures that the brain prioritizes the processing of highly salient and unexpected stimuli at the expense of other ongoing information processing and behaviour [1], [2]. In addition to selecting features that are physically conspicuous and salient [3], the attention circuits support visual sampling of the environment by interacting with motivational and emotional systems [4], [5], [6], [7]. Via these reciprocal links, attention guides our processing resources automatically towards events that are physically dangerous or threatening, such as predatory or poisonous animals [8], threats of violence [9], or facial signals of aggression [10]. These findings have led some researchers to propose that there exists a ‘threat module’ [4], [11] in the brain that tracks the potential danger associated with sensory arrays, and subsequently biases attentional selection towards dangerous targets [4]. But in addition to successful avoidance of danger, an organism's well-being depends crucially on its ability to detect events that could increase its changes for safety and reproduction. Recent studies have indeed revealed that the attentional system also shows selective preference for processing visual information related, for example, to pleasant social interaion[9], [12], [13] and sexual signals [14].

An organism's survival also depends crucially on its ability to maintain steady energy levels. From an evolutionary viewpoint, it would thus be highly beneficial if the cognitive system would also be biased to orient selectively towards *nutrients* in the visual environment. Particularly in environments with high competition for limited nutritional resources, rapid detection of potential sources of energy would facilitate the maintenance of steady energy intake. Several lines of evidence suggest that this might indeed be the case. First, foods are primary reinforcers and have intrinsic hedonic value, and mere sight of food is known to increase the activation of the brain's emotion and reward circuits [15], [16] whose links with the attention systems are well established [4]. Second, prior studies showing that food deprivation enhances attention towards food words [17], [18] suggest that the attentional and reward circuits indeed interact when processing the hedonic value of food-related cues [19]. Third, event-related potential (ERP) studies have demonstrated that the brain constantly tracks the energetic contents of perceived objects: visual images of high and low-calorie foods elicit differential ERPs as soon as 165 ms post stimulus [20], suggesting early differential processing of the hedonic value of foods. Accordingly, it seems likely that evolution could have also shaped the attention circuits for an effective detection of and orienting towards targets with high nutritional value.

But as an individual's energy consumption is significantly influenced by his or her body mass, would it be possible that attentional processing of nutriments is also influenced by the individual's weight? Comparative studies have shown that an interconnected *reward system* comprising of the amygdala, striatal and midbrain regions [21], [22], [23] plays a key role in guiding appetitive as well as addictive behaviours. Feeding and drug use involve learned preferences and habits that have been established by powerful, repeated reinforcing rewards, and the neural circuitry involved in drug addictions and obesity is strikingly similar [24]. Functional neuroimaging studies in humans have revealed that drug-related sensory cues may trigger drug-seeking behaviour by eliciting hyperactivity in the brain's reward circuit, and similarly, food-related cues may trigger food-seeking behaviour via the same system [15], [25]. Reward circuits' exaggerated sensitivity to high-calorie food cues may actually be a critical factor explaining obesity [26], [27], and accordingly, excessive sensitivity to foods in obese individuals has been found to be mediated by hyperactivation of the reward system [28]. Studies using the Stroop task in patients with eating disorders such as bulimia nervosa have established that such individuals have an attentional bias towards food-related word stimuli (see review in ref [29]), whereas eye tracking suggest that patients with anorexia nervosa pay less attention to food pictures than healthy controls [30]. Consequently, it is possible that adaptations in specific neurons in the reward circuit caused by repeated exposure to foods (see e.g. ref. [31]) could also influence reciprocal links between the reward system and attention circuits.

**Figure 1:**
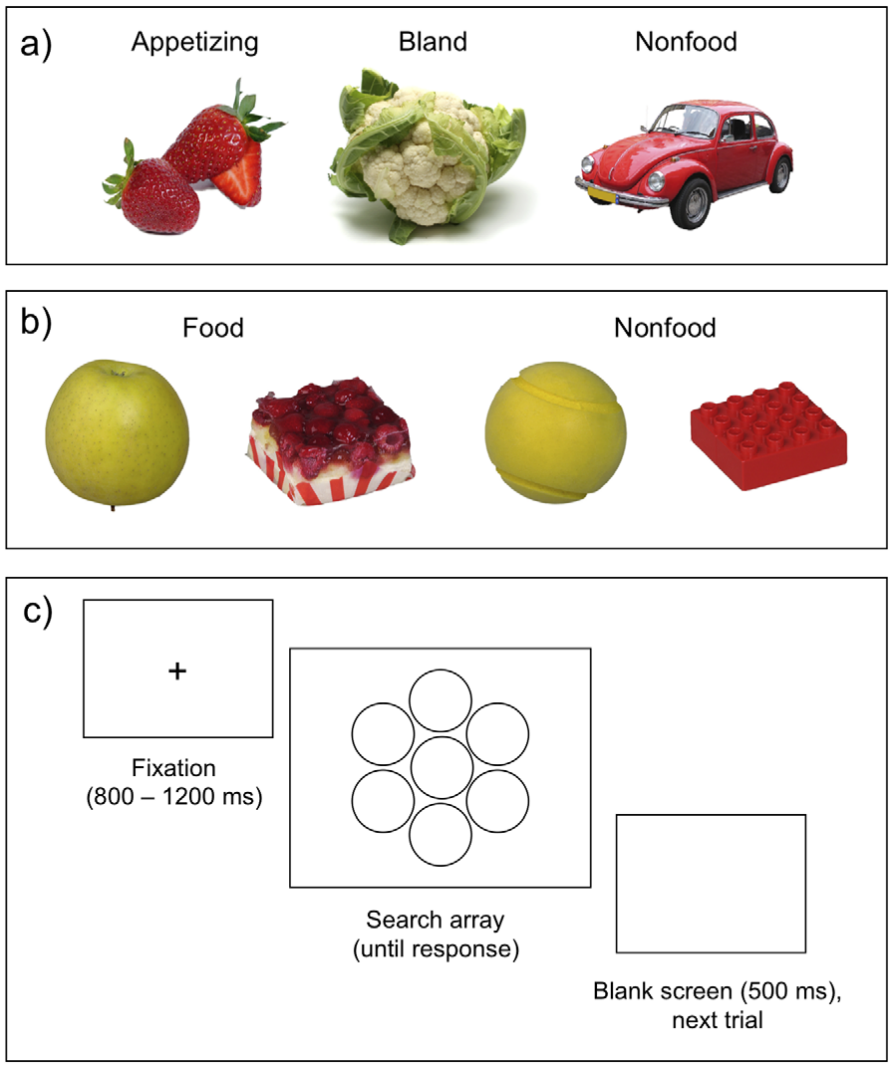
Illustration of the stimuli (a: Experiment 1; b: Experiment 2) and an overview of the experimental procedure (c). In Experiment 1, in target-present trials, the participants searched for appetizing or bland food items embedded in an array of neutral nonfood items (cars) or vice versa; in target-absent trials, all the stimuli belonged to the same category. In Experiment 2, food and nonfood items were matched with respect to shape, colour, and global configuration. Each trial (c) began with a central fixation cross displayed randomly for 800–1200 ms, and was followed by a search array. The array was displayed until the participant responded whether or not it contained a discrepant item, and was followed by a blank screen displayed for 500 ms.

In sum, there is abundant evidence suggesting that the brain is intrinsically prepared for processing the nutritional value of foods once encountered. However, two critical questions remain unanswered. First, do the brain's attentional systems prioritize the visual detection of nutriments? Second, is the detection and selection of food targets modulated by individuals' energy consumption, as indexed by body mass? In the present study we investigated these issues in two visual search experiments. Participants were presented with arrays of food and nonfood items. On half of the trials all stimuli belonged to the same category (either foods or nonfoods), and on another half of the trials there was one discrepant target – either a food target among nonfood distracters or vice versa (see Figure 1). The participants had to decide whether or not there was a discrepant item in the array, and respond with a button press. Response latencies and accuracies were collected. In Experiment 1, we compared the detection speed of both highly appetizing, high-calorie (e.g., chocolate, cakes, pizza) and bland, low-calorie (e.g., lentils, crackers, cabbage) foods among neutral nonfood items (cars) and vice versa. In Experiment 2, we also recorded eye movements and used visually matched food and nonfood targets and distracters, such that participants had to detect, for example, an apple embedded in arrays of tennis balls, a raspberry pie among red LEGO bricks, and so forth. In both experiments, a *food bias* score was computed to reflect the speed advantage (in ms) for detecting food vs. nonfood targets.

Our predictions were straightforward. If the human attentional system is biased towards detection of nutriments, participants should be faster in detecting food items among nonfood distracters than vice versa. Moreover, if the tendency to notice foods is influenced by individual differences in energy consumption, we would expect the food bias scores to correlate with the Body Mass Index (BMI).

## Results

### Experiment 1

**Figure 2:**
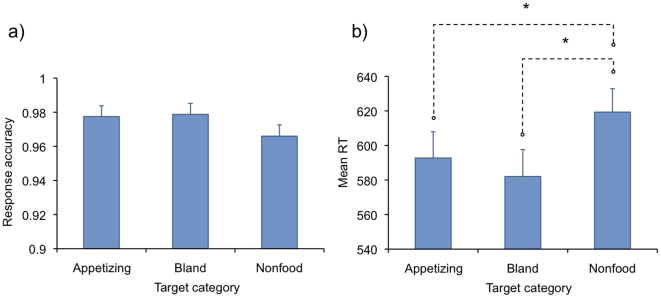
Means and standard errors of the response accuracy (a) and reaction times (b) on target-present trials, as a function of target type in Experiment 1. The asterisk denotes a significant difference (*p*<.05).

The effects of target category (appetizing food vs. bland food vs. non-food) on response accuracy and on response latencies were analyzed separately for the target absent and target present trials by using one-way ANOVAs. See Figure 2 for a summary of the results. For target absent trials, the target category did not influence response accuracy, *F*<1, or RTs, *F* = 1.30. For target present trials, target category did not have an effect on response accuracy either, *F* = 2.40, p = .13, whereas a significant effect was observed on reaction times, *F*(2,52) = 13.78, *p*<.001, η_p_^2^ = .35. Pairwise comparisons (Bonferroni) showed that both appetizing, *t*(26) = 3.32, *p*<.01, and bland, *t*(26) = 6.98, *p*<.001, food targets were detected faster than nonfood targets, but the detection times for appetizing vs. bland food items did not differ from each other, *t*(26) = 1.24, *p* = .23. These effects remained significant even when self-reported hunger scores were used as a covariate in the analyses.

**Figure 3:**
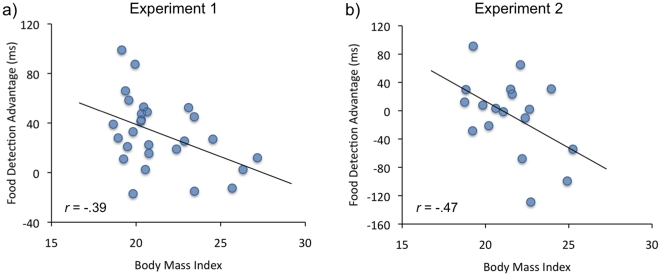
Linear negative association between food detection bias in manual response latencies and Body Mass Index in Experiment 1 (a) and 2 (b). The black line shows the least-square regression line.

Next, we computed a *food detection advantage* score by subtracting the mean RT to food targets from the mean RT to nonfood targets, separately for appetizing and bland foods as well as for all foods together. Positive bias scores reflect bias towards food and negative towards nonfood targets. A significant negative correlation between overall food detection advantage and BMI was found, *r* = −.39, *p* = .04, with lower BMI scores resulting in an advantage—and higher scores in a disadvantage—in detecting food targets among nonfood distracters (Figure 3a). A similar bias was observed towards both appetizing, *r* = −.36, *p* = .02 and bland, *r* = −.32, *p* = .05 (one-tailed) food items. Detection advantage for appetizing over bland foods did not correlate significantly with BMI, *p*>.05. Self-reported hunger at the time of the experimental session correlated neither with BMI nor with the food detection advantage, *p*s>.05.

The analysis of the visual saliency scores revealed significant differences between stimulus categories, F(2,117) = 14.49, *p*<.001, η_p_^2^ = .20 (*M*_appetizing_ = 37.36, *M*_bland_ = 37.58, *M*_nonfood_ = 55.43). Planned comparisons revealed that saliency scores were higher for the nonfood versus appetizing, *t*(88) = 4.80, *p*<.001, and bland, *t*(88) = 4.313, *p*<.001, items, whereas no significant difference was observed between appetizing and bland items, *t*(58) = 0.41, *p* = .98.

Participants rated the emotional valence of the food and nonfood items after the experiment. The mean (standard errors in parentheses) valence scores (*M*_appetizing_ = 6.64 (.18), *M*_bland_ = 3.96 (.24), *M*_nonfood_ = 4.41 (.25)) differed between the categories, *F*(2,52) = 38.49, *p*<.001, η_p_^2^ = .60, and planned comparisons revealed that appetizing foods were rated as more pleasant than bland foods, *t*(26) = 10.23, *p*<.001, or nonfood items *t*(26) = 7.60, *p*<.001, whereas there was no difference between the valence ratings for bland and nonfood items, *t*(26)<1, *p* = .33. Subjective valence ratings of appetizing, bland and nonfood items did not correlate with the detection times for the target absent or target present trials, all *p*s>.05.

### Experiment 2

In Experiment 1 the food and nonfood items were visually heterogeneous. An elaborate computational modelling of the visual saliency of the stimulus arrays showed that the food items were, in fact, visually *less* salient than the non-food items. Therefore, the detection advantage for food items is not easily explained by saliency differences between food and non-food items. However, it is still possible that some low-level visual factors intrinsically associated with foods but not captured by the available low-level image statistics or visual saliency scores could explain the food detection advantage. Additionally, only manual responses were acquired making it impossible to conclude at which cognitive processing stage the food detection bias occurred. To overcome these limitations, Experiment 2 involved visually matched food and nonfood stimuli, as well as eye movement recordings during the visual search task. As no visual search times for appetizing vs. bland foods did not differ in Experiment 1, this distinction was not further explored in Experiment 2.

The visual search process (i.e., time from search array onset to manual response) was divided into two stages using the eye movement data [32]. *Attentional orienting time* was defined as the time from the onset of the stimulus display until the target item was initially fixated. Detection efficiency was assessed by *decision time*, that is, the time after the target was initially fixated until the manual response. Additionally, mean dwell times for the food and nonfood items were computed. Food advantage was computed for all these eye movement metrics.

Now that the food and nonfood items were visually homogenous, no overall food detection bias was observed. Although first fixations landed early on both the discrepant food and nonfood targets (Mean latencies: *M*_foods_ = 378 ms, *M*_nonfoods_ = 360 ms), none of the manual response or eye movement measures were influenced by target type either for the target present or target absent trials, *F*s<1.60. Nevertheless, it was again found that BMI showed a significant negative correlation with food search advantage scores, both for the latency of first fixation on target (attentional orienting time), *r* = −.40, *p* = .05 (one-tailed), as well as for the manual response latency (Figure 3b), *r* = −.47, *p* = .04. Importantly, BMI was not significantly correlated with the bias score for recognition (i.e., time from 1^st^ fixation on target until response), *p* = .18. This suggests that the BMI-contingent bias is due to differences in the speed of selective attentional orienting to food vs. nonfood items, rather than differences in processing the targets once fixated. Self-reported hunger correlated neither with BMI nor with the food detection advantage, *p*s>.05. The analysis of the visual saliency scores revealed that visual saliency was slightly higher (mean difference of 7.77 units) for food targets embedded in nonfood arrays versus nonfood targets embedded in food arrays, t(94) = 2.70, *p* = .01.

## Discussion

The present results show that the human visual system is specialized in detecting nutriments among other visual objects in cluttered visual environments. In Experiment 1, both appetizing and bland foods were detected faster among nonfood items than vice versa, which suggests that evolution has shaped the attention for effective detection of visual features that signal opportunities for energy intake. Prior studies have suggested that motivational states related to food influence attention towards food-related items. In line with this, functional MRI studies have established that hunger boosts both the reward [33] and the attention [19] circuits' responses to pictures of foods, and behavioural data show that hungry individuals spend more time than satiated individuals in attending to food words in an attentional dot-probe task [17], [18]. We extend these findings by showing that already the attentional *detection* of food vs. nonfood items is prioritized in the visual system as indexed by rapid detection of food targets among nonfood distracters. Such specialization is biologically highly plausible, as it facilitates energy intake by making nutritious objects ‘pop out’ from cluttered environments.

In general, the present results are in line with the data showing that attention does not only monitor and respond to signals of threat [4], [11], but also towards rewarding and pleasant events [9], [32], [34]. However, our data from Experiment 1 go beyond an observation that the mere hedonic value (appetizing/pleasant vs. nonappetizing/neutral) associated with the foods would be responsible for the attentional bias. Quite the contrary, the data imply that the stimulus *category* (food vs. nonfood) was the feature that governed attentional deployment (c.f. refs. [35], [36]): Both appetizing and bland food targets were detected faster than nonfood targets but there was no difference in detecting appetizing vs. bland foods. And importantly, only appetizing but not bland foods were rated as more pleasant than the control objects, hence an elevated valence or pleasantness of food items is unlikely to explain the results. Accordingly, the critical feature influencing orienting of attention was whether the target object provided nutrition or not, rather than whether it was perceived as more pleasant than its surroundings. In line with this, functional imaging studies [28] have shown that even non-appetizing (or low-calorie) foods engage the reward circuit more strongly than nonfood items such as cars. Altogether these findings show how the reciprocal links between the object recognition, emotion and attention systems may be tuned very narrowly to respond to specific features (here the nutritional value) of the visual stimuli [37]: after a certain threshold of nutritional value is detected, the system triggers an attention shift towards the object no matter how much energy the object actually contains. Importantly, computational modelling of the visual saliency of the search arrays in Experiment 1 revealed that this effect cannot be attributed to simple sensory properties of foods, because food items were visually *less* salient than nonfood items.

Although Experiment 1 established strong attention bias towards foods, this bias disappeared when food and nonfood items were visually strictly matched in Experiment 2. The most obvious explanation for this effect is that specific visual features or cues such as shape-color combinations have been associated with certain foods, and rapid detection of these cues can serve as a short-cut for peripheral detection of food items (see refs. [13], [37]). When these features become highly familiar, they may start to guide attention in automatic fashion [38], [39] even though the visual features associated with foods would not be visually highly salient (c.f. saliency data from Experiment 1). But when the nonfood distracter items convey similar shape-color cues that have originally been associated with foods (c.f. Experiment 2), the visual system can no longer rapidly distinguish foods from nonfoods and the attentional bias towards food items disappears, as was the case in Experiment 2. This view is further supported by the finding that there was no overall bias towards food items in experiment 2, even though the foods were visually slightly more conspicuous than the nonfood items. Accordingly, some complex visual features associated with foods rather than their mere visual saliency must guide the food detection bias in Experiment 1.

### Food Bias and BMI

The most striking finding of Experiments 1–2 was that the detection bias towards food versus nonfood items was negatively associated with participants' BMI. In Experiment 1 with visually dissimilar food and nonfood items, 89% of the subjects showed faster detection of foods than nonfoods, but this detection bias was stronger among the more lean individuals. However, when food and nonfood items were visually matched in Experiment 2, no overall bias towards foods was observed in the data. Given that visual similarity between targets and distracters is a major determinant of visual search performance [40], this finding is not too surprising. However, more importantly, despite the lack of an overall bias in these conditions, we again found that search performance was negatively correlated with BMI: Those with lowest BMI showed food detection *advantage* and those with highest BMI showed a food detection *disadvantage*.

On the basis of the manual response data (Experiment 1), it is not possible to determine at which cognitive stage this attentional bias occurs. For example, it is equally likely that BMI would be associated with the speed of attentional orienting or with the time taken to decide that a target is discrepant once it has been visually attended. However, the eye movement data of Experiment 2 allowed us to decompose the visual search process into separate *orienting* and *decision* stages, and to assess at which stage the bias occurs. These analyses confirmed that BMI was specifically associated with attentional *orienting*, as *o*nly the latency of the initial fixation on the discrepant target was significantly correlated with BMI. However, a similar association was not observed for decision times (i.e., time from the 1^st^ fixation on the target to the manual response), which shows that the actual decision making process (i.e., deciding whether the target was discrepant or not) was not related to BMI.

But what kinds of mechanisms ultimately cause this BMI-contingent attentional bias? As eating involves learned preferences and habits that have been established by powerful, repeated reinforcing rewards, it is possible that the eating history of an individual would tune the attentional and reward systems to be selectively responsive to certain types of foods. The present data could suggest that such tuning is most narrow among the most lean individuals, as i) they showed the largest bias towards foods in Experiment 1, and ii) they showed such a bias in Experiment 2 even when the food and nonfood items were visually similar. Accordingly, the individuals with smallest BMIs were successful in discriminating foods from nonfoods, even though they were visually similar to the surrounding nonfood items. However, the individuals with higher BMIs might have more broadly tuned representations of potential nutritional items, which makes their visual search process less efficient, particularly under more discrimination-demanding conditions. This selective tuning hypothesis is also supported by prior eye tracking studies showing that, for example, individuals with anorexia nervosa pay less attention to foods than healthy controls [30].

Given that the reward circuit in the brain shows elevated responses to mere visual perception of foods [15], [16] and that this circuit interacts with the attention systems during the perception of food targets [19], it can be speculated that individual differences in the reward circuit's responses to foods could account for the observed effects. Positron emission tomography (PET) studies targeting the neurochemistry of the reward circuit provide corroborating evidence for this view. Food consumption is associated with dopamine release in the dorsal striatum in healthy subjects [41], and the baseline type 2 dopamine receptor (D_2_R) density is inversely proportional to BMI [42]. It has been proposed that this lowered D_2_R density in obese individuals could represent downregulation to compensate for transient dopamine increases due to perpetual overstimulation of the reward circuit by eating [42]. Accordingly, such blunted signalling in the dopaminergic link of the reward circuit might make individuals with higher BMIs less likely to detect the nutritional value of food when it is initially encountered, which could lead to slower detection of food targets in visual search tasks.

### Limitations of the study

Although the two experiments reported here provide novel evidence regarding attentional bias towards nutrients, certain limitations should be borne in mind when interpreting the relevance of the findings. First, we correlated only a relatively simplistic proxy (BMI) of body metabolism and eating behaviours with the food detection advantage, thus we cannot pinpoint the specific mechanism that results in the BMI-contingent attentional bias. Second, the study was conducted with healthy university students with a narrow range of BMI scores, hence the results might not be extrapolated to patient populations. For example, whereas we observed a negative correlation between BMI and food detection bias, studies of individuals with eating disorders such as bulimia nervosa show a positive attentional bias towards food stimuli [29]. Accordingly, it is possible that the actual association between BMI and food detection bias would be u-shaped when the full range of BMI scores is considered: within the typical BMI range the association seems to be negative (c.f. this study), but after a certain threshold value of BMI the association would be rendered positive. This speculation needs to be confirmed in future studies with participants with a more extended range of BMI scores. Finally, it must be stressed that we cannot draw strong conclusions regarding the causal relationship between food bias and BMI – it is possible that individual differences in food bias could be a risk factor for gaining weight, but it is equally likely that increased weight could modify an individual's visual biases towards foods. Longitudinal studies on the effects of weight loss or weight gain on the food detection bias are thus required to reveal the exact causal link between food detection bias and obesity.

### Conclusions

Maintaining steady energy levels is essential for an organism's effective functioning, and the human cognitive system has evolved to facilitate energy intake by biasing the visual attention selectively towards targets that may provide nutrition. However, such attentional processing is modulated by individuals' body mass. Such differences in the ability to detect and localize nutriments in the environment may ultimately be one potential risk factor for obesity. Although highly speculative, the present data thus suggest that individuals who are effective in detecting nutrients in the environment do not need to stockpile energy resources upon detecting them, and may not be at risk for obesity. On the contrary, those whose attention is less biased towards detecting nutrition may need to consume foods upon sight. In the present-day wealthy societies with practically unrestricted access to food, such behaviour might result in weight gain and ultimately in obesity.

## Materials and Methods

### Ethics Statement

The research was conducted according to the ethical standards of the American Psychological Association (APA). According to Finnish regulations, specific ethics approval was not necessary for this study.

### Participants

Twenty-seven (4 males) volunteer students with a mean age of 25 (*SD* = 4.1) from the University of Turku participated in Experiment 1, and eighteen different volunteers (4 males, mean age of 24 years, *SD* = 3.1) from the same student pool in Experiment 2. All gave written informed consent. Before the experiment, they reported body mass and height, and gave a self-report of their hunger level using a visual analogue scale ranging from 0 to 100. All participants had corrected or corrected to normal vision, and none were obese (*M*_BMI_ = 21, *SD*_BMI_ = 1.9).

### Stimuli and Experimental Design

The stimuli and the experimental design are summarized in Figure 1. In Experiment 1, the stimuli were full-colour pictures of thirty appetizing (e.g., strawberries, pizza, fruit pie) and thirty bland (e.g. cabbage, lentils, potatoes) food items, and sixty nonfood items (cars). In Experiment 2, the stimuli were 48 full-colour pictures (selected from ref [43]) of food items and 48 visually matched nonfood items. The food pictures contained both high and low calorie items. Each food item was paired with a visually similar nonfood item (e.g., apple – tennis ball and raspberry pie – red LEGO brick), and each target present trial contained the target embedded within a matrix of the corresponding matched items. In both experiments, the stimulus categories were equated with respect to mean luminance, SD of luminance distribution, and contrast density (RMS contrast), as assessed by means of MATLAB 7.0 (The MathWorks, Natick, MA).

The stimuli were shown on a 21″ monitor (120 Hz refresh rate) with a 3.2 GHz Pentium IV computer. They were presented either in a circular array of 7 stimuli (Experiment 1) or in a 3×3 rectangular array without a central stimulus (Experiment 2), with the average distance between the fixation point at the center of the screen and the inner edge of stimuli being 4.5° (Experiment 1) or 5.25° (Experiment 2). The targets never appeared at the central position. Each trial began with a central fixation cross displayed randomly for 800–1200 ms, and in Experiment 2 the trial was not initiated before the participant was actually fixating the cross. After that, a search array was presented, and the participants' task was to decide as quickly and accurately as possible whether or not the array contained a discrepant item by pressing one of two pre-specified keys. After the response, a blank screen was displayed for 500 ms and the next trial was initiated. There were four types of trials: (i) food targets with nonfood distracters, (ii) nonfood targets with food distracters, (iii) only food items and (iv) only nonfood items. In Experiment 1, half of the trials containing food targets had appetizing items and half had bland food items. Experiment 1 had 30 trials of each type and Experiment 2 had 48 trials of each type. Both experiments were split into two blocks and began with ten practice trials.

In both experiments, visual search performance was assessed by participant-wise (a) response accuracy and (b) mean response latency (values below 80 ms and 2SDs above the participant's mean across all conditions were removed) measured from the onset of the stimulus display until the participant responded whether there was a discrepant item or not. After the experiment, the participants rated the valence of the food and nonfood items using a self-assessment manikin [44].

### Computational modelling of visual saliency

Various models posit that visual saliency influences initial shifts of covert and overt attention (see ref. [45]). The evidence in support of these models demonstrate that the distribution of initial eye fixations on a picture is determined by the saliency weights of the different parts of the image [46], [47]. To control for such low-level differences between the food and nonfood targets the search arrays, a saliency map was computed for each array of appetizing or bland food targets among nonfood distracters, and vice versa (Experiment 1) or food targets among nonfood distracters and vice versa (Experiment 2). The iNVT Neuromorphic Vision Toolkit (see ref [3]) was used for modelling the visual saliency of the search arrays. The resulting map identifies the saliency of each pixel in an image on the basis of variations in orientation, intensity, color, and their combinations. Salient areas or objects thus stand out from the background, including other surrounding objects. The program's default weightings were employed to avoid prioritization of any of the three dimensions. Subsequently, saliency scores were computed for targets and distracters in each stimulus array.

### Eye movement recordings

In Experiment 2, participants' eye movements were recorded with an EyeLink II eyetracker (SR Research, Mississauga, Ontario, Canada) connected to a 2.8 GHz Pentium IV computer. The sampling rate of the eyetracker was 500 Hz, and the spatial accuracy was better than 0.5°, with a 0.01° resolution in the pupil-tracking mode. A nine-point calibration and validation was completed prior to the experiment, and drift correction was performed at the beginning of each trial. Rectangular regions of interest were drawn around each location where the stimuli could appear in the screen. Prior to data analyses, anticipatory eye movements (latencies below 80 ms) were discarded.
